# Genome Sequences of Mycobacteriophages Joselito, Patt, and Tydolla

**DOI:** 10.1128/MRA.00519-20

**Published:** 2020-06-04

**Authors:** Christina Jacob, Yunia Alvarez, Tara Carney, Cynthia Castro, Regina Alvarez, Bernadette J. Connors

**Affiliations:** a Department of Science, Dominican College of Blauvelt, Orangeburg, New York, USA; DOE Joint Genome Institute

## Abstract

Mycobacteriophages Joselito, Patt, and Tydolla were isolated from different soil or water samples using Mycobacterium smegmatis mc^2^155 as the host. Each was obtained using direct isolation techniques, purified, and then sequenced using the deconvolution of genomes after *en masse* sequencing (DOGEMS) method.

## ANNOUNCEMENT

Phages Joselito, Patt, and Tydolla were directly isolated from soil collected near a body of water (Joselito and Patt) or from a landscaped flower bed (Tydolla). These phages were isolated using Mycobacterium smegmatis mc^2^155 as the host and cultured on 7H9 medium containing 1 mM CaCl_2_ at 37°C. This bacterial strain has served as a useful host for mycobacteriophage isolation as part of the Science Education Alliance–Phage Hunters Advancing Genomics and Evolutionary Science (SEA-PHAGES) program, which has curated over 1,900 sequenced mycobacteriophage genomes (1). After two rounds of plaque purification, DNA was purified from a high-titer lysate using the Promega Wizard DNA cleanup kit. Genomic DNA was pooled before library preparation, and sequencing libraries were prepared from 48 phages using the Ultra II FS kit with dual-indexed barcoding (New England Biolabs). The libraries were then run on an Illumina MiSeq instrument, yielding at least 46-fold coverage for each genome, determined by aligning all reads to complete genomes and checking the read depth. The assembly was completed on raw reads using default parameters for Newbler 2.9, and single-phage contigs were produced (2). These sequences were checked for accuracy and genomic termini using Consed version 29 (3). The resultant contigs were identified after the sequences were subjected to BLASTN searches on PhagesDB. The phage to which the longest contigs belonged was determined by PCR using primers that spanned two putative open reading frames in each phage contig. The genomes were then manually annotated using DNA Master (4) following autoannotation with Glimmer (5) and GeneMark (6). Functions were predicted using BLASTp on the NCBI nr and actinobacteriophage databases (E value, 10e-7 or less), as well as HHPRED (probability value, 90% or higher, if the query aligned the full length of the subject match, or if the alignment included the functional domain) (7–9). Functional annotation based on synteny was determined using Phamerator (10), and ARAGORN and tRNAScan were used to detect tRNAs (11, 12). The phage genome features and assembly results are listed in Table 1.

**TABLE 1:** Mycobacteriophage genome details and assembly results

Phage	GPS coordinates	Accession no.	SRA accession no.	Length (bp)	GC content (%)	No. of CDS^*a*^	Coverage (×)/no. of library reads	Primer sequences	
								Forward	Reverse
Joselito	41°03'10.1"N, 73°57'00.4"W	MH338237	SRX7690244	52,347	63.7	98	92/850,740	GAGATCGCGTGCGTCACC	CCAGTACGCCTCAAGGTCTACG
Patt	41°02'49.9"N, 73°57'02.1"W	MK524488	SRX7690246	54,611	67.6	88	46/978,452	CAATGGCAGCGATCGGCCT	GTCGTCGAGGGTGAGCTTGTCGA
Tydolla	41°02'49.9"N, 73°57'02.1"W	MH576977	SRX7690250	68,657	67.5	99	89/978,449	GACCCTACCTGCGGAACGCA	TGCGGAGTCGGCTTCGATC

a CDS, coding DNA sequences.

Clusters and subclusters are determined by using nucleotide similarity to other phage genomes (13). Patt (subcluster K4) and Joselito (subcluster A11) showed genome-wide similarity to the other temperate phages in their respective subclusters, sharing 98% and 99% identities with their closest relatives, Reptar3000 (GenBank accession number MH926058.1) and Munch (GenBank accession number MK524513.1), respectively. Tydolla (subcluster B3) had 99% identity to other lytic phages, such as Chandler (GenBank accession number KP027207.2). Negative-staining transmission electron microscopy showed that these mycobacteriophages have siphoviral morphology, which is expected of viruses in these subclusters (images available at https://phagesdb.org/).

These genomes contain genes associated with phage structure and assembly. Terminases, portal proteins, major capsid proteins, major and minor tail proteins, and tape measure proteins are present, found in the 5′ arm of the three genomes. Lysis cassettes comprised of lysin A, lysin B, and holin genes are also found, although the organization of these genes is different among these phages. The temperate phages also have genes associated with the formation of lysogens (integrase and immunity repressor).

### Data availability.

The genome sequences reported here have been deposited in GenBank under the accession numbers in Table 1 (BioProject accession number PRJNA488469). The SRA numbers are also provided in Table 1.
